# Melatonin mitigates cadmium phytotoxicity through modulation of phytochelatins biosynthesis, vacuolar sequestration, and antioxidant potential in *Solanum lycopersicum* L

**DOI:** 10.3389/fpls.2015.00601

**Published:** 2015-08-11

**Authors:** Md. Kamrul Hasan, Golam Jalal Ahammed, Lingling Yin, Kai Shi, Xiaojian Xia, Yanhong Zhou, Jingquan Yu, Jie Zhou

**Affiliations:** ^1^Department of Horticulture, Zhejiang University, HangzhouChina; ^2^Zhejiang Provincial Key Laboratory of Horticultural Plant Integrative BiologyHangzhou, China; ^3^Key Laboratory of Horticultural Plants Growth, Development and Quality Improvement, Agricultural Ministry of ChinaHangzhou, China

**Keywords:** cadmium, food safety, melatonin, phytochelatins, tomato, vacuolar sequestration

## Abstract

Melatonin is a ubiquitous signal molecule, playing crucial roles in plant growth and stress tolerance. Recently, toxic metal cadmium (Cd) has been reported to regulate melatonin content in rice; however, the function of melatonin under Cd stress, particularly in higher plants, still remains elusive. Here, we show that optimal dose of melatonin could effectively ameliorate Cd-induced phytotoxicity in tomato. The contents of Cd and melatonin were gradually increased over time under Cd stress. However, such increase in endogenous melatonin was incapable to reverse detrimental effects of Cd. Meanwhile, supplementation with melatonin conferred Cd tolerance as evident by plant biomass and photosynthesis. In addition to notable increase in antioxidant enzymes activity, melatonin-induced Cd stress mitigation was closely associated with enhanced H^+^-ATPase activity and the contents of glutathione and phytochelatins. Although exogenous melatonin had no effect on root Cd content, it significantly reduced leaf Cd content, indicating its role in Cd transport. Analysis of Cd in different subcellular compartments revealed that melatonin increased cell wall and vacuolar fractions of Cd. Our results suggest that melatonin-induced enhancements in antioxidant potential, phytochelatins biosynthesis and subsequent Cd sequestration might play a critical role in plant tolerance to Cd. Such a mechanism may have potential implication in safe food production.

## Introduction

The rising concern on cadmium (Cd) as a toxic heavy metal, is not only due to its detrimental effects on crop production, but also for potential health hazards associated with food chain contamination (Hall, 2002; Mobin and Khan, 2007; Ajjimaporn et al., 2012). Given that Cd is not an essential nutrient, but easily assimilated in plants due to flexible specificity of ion channels and divalent metal transporters (Liu et al., 2003). Upon accumulation, Cd disrupts normal metabolism leading to diverse morphological, physiological, biochemical and cellular changes (Llamas et al., 2000; Pietrini et al., 2003). Inhibition of Calvin cycle enzymes, photosynthesis, and carbohydrate metabolism by Cd results in low biomass accumulation in plants (Mobin and Khan, 2007). Although Cd does not participate in Fenton-reaction, it induces reactive oxygen species (ROS) accumulation and eventually causes oxidative stress in plant cells (Romero-Puertas et al., 2002; Pietrini et al., 2003; Cui et al., 2012). Moreover, Cd stress alters plasma membrane permeability by inhibiting H^+^-ATPase, which plays important roles in ionic balance and substance transport (Janicka-Russak et al., 2012). At cellular level, Cd alters plant cell cycle and division, and increases chromosomal aberrations and malformed embryos (DalCorso et al., 2010). Cd contamination severely limits crop production particularly in marginal areas of developing countries, where cultivation is being injudiciously expanded to meet rising demand of food. On the other hand, Cd accumulation in edible plant parts ultimately poses threat to human health as Cd toxicity is closely associated with various health hazards including kidney damage, bladder and lung cancer (Ajjimaporn et al., 2012). Therefore, the enhancement of Cd tolerance in plant to boost crop yield in marginal area as well as reduction of Cd content in edible plant parts for food safety, are ongoing endeavors in plant science and food production.

Plants have intrinsically evolved sophisticated cellular mechanisms for Cd detoxification and tolerance such as immobilization, exclusion, chelation, compartmentalization of metal ions and the repair of cell structural alteration (di Toppi and Gabbrielli, 1999; Hall, 2002). The root cell wall, which is in direct contact with heavy metals in soil, can first release chelating compounds, such as phytosiderophores, nicotianamine and organic acids, to bind Cd and other heavy metals in the epidermis (Xiong et al., 2009). Plant cells can increase the leakage of solutes and reduce influx across plasma membranes to exclude Cd and other heavy metals (Meharg, 1993). Moreover, heat shock proteins and metallothioneins have been reported to repair and protect plasma membranes under heavy metal stress (Neumann et al., 1994; Hall, 2002). The chelation of heavy metals with high-affinity ligands in the cytoplasm and its subsequent compartmentalization in the vacuole are generally considered as the “first line” defense mechanisms in plant Cd tolerance (Hall, 2002; Park et al., 2012). Phytochelatins (PCs) comprise a family of peptides (γ-Glu-Cys)n-Gly (*n* = 2–11) that are synthesized from glutathione (GSH). PCs synthesis is rapidly induced by Cd treatment that chelate Cd and sequester the PC-Cd conjugates into the vacuoles (Rauser, 1995). Besides, GSH activates antioxidant enzymes such as superoxide dismutase (SOD), ascorbic acid peroxidase (APX), and catalase (CAT) that neutralize Cd-induced ROS for cellular redox homeostasis (Jozefczak et al., 2014).

In the past decade, the complex molecular mechanisms of Cd stress response have been extensively elucidated in plants (DalCorso et al., 2010). Involvement of several phytohormones such as abscisic acid (ABA), brassinosteroids (BRs), jasmonic acid (JA), ethylene (EHT), and salicylic acid (SA) was found to be associated with Cd stress response (Metwally et al., 2003; Maksymiec, 2007; Masood et al., 2012; Ahammed et al., 2013; Stroinski et al., 2013). Previously, we found that exogenous 24-epibrassinolide (EBR, a biologically active BR) could not only alleviate Cd-induced photosynthetic inhibition and oxidative stress, but also could reduce Cd content in tomato plants (Ahammed et al., 2013). Considering the hazardous effects of Cd on plants and animals, extensive researches are still underway to explore new natural compounds which can improve plant tolerance to Cd and/or minimize toxic Cd content in edible plant parts for food safety purpose.

Most recently, Cd has been reported to regulate melatonin content in rice (*Oryza sativa*), a ubiquitous signal molecule, well-accepted as a new plant growth regulator and/or biostimulator rather than plant hormone (Arnao and Hernández-Ruiz, 2014; Byeon et al., 2015). Melatonin (*N*-acetyl-5-methoxytryptamine) has long been known to be an important animal hormone involved in multiple biological processes (Escames et al., 2012; Calvo et al., 2013). Although its function as a hormone has been well-characterized in animals, our knowledge in plant kingdom is still fragmentary (Arnao and Hernández-Ruiz, 2014). In recent years, melatonin appeared as a research focus in plant biology. Melatonin is considered as a plant growth regulator because of its specific physiological functions such as regulation of the growth of roots, shoots and explants, seed germination, rhizogenesis and delay in leaf senescence (Arnao and Hernández-Ruiz, 2014). In addition, melatonin has been found to fortify plant tolerance to a range of abiotic stresses such as drought, cold, heat, salinity, chemical pollutants, herbicides, and UV radiation (Zhang et al., 2015). The mechanisms of melatonin-mediated stress tolerance involve the promotion of antioxidant biosynthesis, the activation of related enzymes and the direct scavenging of ROS following the exposure of plants to harsh environments (Li et al., 2012; Tan et al., 2012; Park et al., 2013; Zhang et al., 2013; Bajwa et al., 2014). Like any other abiotic stress, heavy metals such as Cd significantly induces melatonin content in lower as well as higher plant species. Strikingly, exogenous melatonin can ameliorate Cd-induced stress in green micro algae; however, its role in higher plants still remains elusive. Although PCs are well known to bind many toxic metals including Cd to promote metal detoxification and subsequent stress tolerance in plants, information relating to melatonin-induced PC biosynthesis and/or stress tolerance in higher plants is missing.

To better understand the mechanistic basis of melatonin in plant stress responses, we sought to identify and functionally analyze melatonin under Cd stress. We hypothesize that apart from its well-known antioxidative and chelating properties, melatonin might have a protective role in Cd tolerance involving other mechanisms. We consider PC biosynthesis as a novel target for melatonin-mediated Cd stress response. To testify this hypothesis, we carried out a set of experiments in tomato (*Solanum lycopersicum* L.) taking photosynthetic function and ROS generation as biomarkers. This study explored crucial mechanisms involved in melatonin-mediated Cd stress response which might have potential implication for ensuring food safety and food security in marginal agriculture.

## Materials and Methods

### Plant Materials and Experimental Design

Tomato seeds (*Solanum lycopersicum* L. cv. Ailsa Craig) were germinated in a growth medium containing a mixture of vermiculite and perlite (3:1, v/v) in a greenhouse. When the first true leaves were fully expanded, a group of six uniform seedlings were transferred into a container (40 cm × 25 cm × 15 cm) filled with Hoagland’s nutrient solution for hydroponics culture. The growth conditions were as follows: a temperature of 25/17°C (day/night), a mean relative humidity of 80%, a photosynthetic photon flux density (PPFD) of 800 μmol m^-2^ s^-1^, and a photoperiod of 14/10 h (day/night).

To investigate the influence of Cd on endogenous melatonin content, the roots of tomato seedlings, at the four-leaf stage were treated with 25 and 100 μM Cd in hydroponics. Cd was supplied in the form of CdCl_2_ (analytical grade). Based on our preliminary experiments, 100 μM Cd was selected for studying effects of exogenous melatonin on Cd stress response. Foliar portions of tomato plants were sprayed with 0, 25, 50, 100, 250, and 500 μM melatonin. Hoagland’s nutrient solution with or without Cd, were changed at every 5 days followed by foliar spray of melatonin. The experiment was terminated 2 weeks after initial Cd treatment. Four replicates were used for each treatment, and each replicate consisted of 12 plants.

### Gas Exchange and Chlorophyll Fluorescence Measurements

Net photosynthetic rate (*P*n) and the maximum quantum efficiency of photosystem II photochemistry (*F*v/*F*m) were determined in the second fully expanded leaves from the plant tops. The *P*n was determined using an infrared gas analyzer (IRGA) portable photosynthesis system (LI-COR 6400, Lincoln, NE, USA). The air temperature, air relative humidity, CO_2_ concentration, and PPFD for *P*n measurement were 25°C, 85%, 400 μmol mol^-1^, and 1000 μmol m^-2^ s^-1^, respectively. The *F*v/*F*m was determined after 30 min of dark adaptation using an imaging pulse amplitude-modulated (PAM) fluorimeter (IMAG-MAXI; Heinz Walz, Effeltrich, Germany) as described (Zhou et al., 2014).

### Determination of H_2_O_2_, Lipid Peroxidation and Electrolyte Leakage

The H_2_O_2_ and MDA concentrations in leaves and roots were assessed spectrophotometrically. To determine the H_2_O_2_ concentration, 0.3 g of fresh sample was homogenized in 3 mL of pre-cooled HClO_4_ (1.0 M) using a pre-chilled mortar and pestle (Willekens et al., 1997). The homogenates were then transferred to 10-mL plastic tubes and centrifuged at 6000 *g* for 5 min at 4°C. The pH of resulting supernatant’s was adjusted to 6.0 with 4 M KOH and centrifuged at 6000 *g* for 1 min at 4°C. The resulting supernatant was passed through an AG1.8 prepacked column (Bio-Rad, Hercules, CA, USA) and H_2_O_2_ was eluted with double-distilled H_2_O. The sample (800 μL) was mixed with 400 μL reaction buffer containing 4 mM 2,2′-azino-di(3-ethylbenzthiazoline-6-sulfonic acid) and 100 mM potassium acetate at pH 4.4, 400 μL deionized water, and 0.25 U of horseradish peroxidase. Equal recovery from the different samples was checked by analyzing duplicate samples. The lipid peroxidation level was determined by quantifying the MDA equivalents using 2-thiobarbituric acid (TBA), as described by Hodges et al. (1999). In brief, samples (0.5 g) were homogenized in 5 mL 0.1% (w/v) trichloroacetic acid (TCA). The homogenates were centrifuged at 10,000 *g* for 10 min, and 4 mL 20% TCA containing 0.65% (w/v) TBA was added to 1 mL of the supernatants. The mixtures were heated in a hot water bath at 95°C for 25 min and immediately placed in an ice bath to stop the reaction. They were then centrifuged at 3000 *g* for 10 min, and the absorbance of the supernatants was recorded at 440, 532, and 600 nm. The MDA equivalents were calculated as described previously (Hodges et al., 1999). To the measurements of electrolyte leakage (EL), plant samples were cut into small pieces with razor blade, rinsed briefly with deionized water, and immediately placed in a tube with 5 mL of deionized water. EL was then measured before and after 4 h of rehydration and ultimately after autoclaving according to the method described by Bajji et al. (2002).

### Histochemical Staining of H_2_O_2_ and $O_{2}^{•-}$ in Leaves

H_2_O_2_ accumulation in leaves was visually detected by staining with 3, 3-diaminobenzidine (DAB) using the method as described (ThordalChristensen et al., 1997). Leaves were immediately removed from plants, submerged in DAB solution (1 mg mL^-1^, pH 3.8) and incubated under light for 6 h at 25°C. $O_{2}^{•-}$ was detected with the nitroblue tetrazolium (NBT) staining procedure, which involved the incubation of the leaves with NBT (0.5 mg mL^-1^, pH 7.8) solution in the dark (Jabs et al., 1996).

### Extraction and Assay of Antioxidant Enzymes

For enzyme extraction, 0.3 g of fresh sample was homogenized in 50 mM potassium phosphate buffer (pH 7.0) containing 0.1 mM EDTA and 1% polyvinylpyrrolidone (w/v). The homogenate was centrifuged at 12,000 *g* for 15 min at 4°C, and the supernatant was collected. This supernatant was used to measure the activities of SOD, guaiacol peroxidase (G-POD), APX, CAT, and glutathione reductase (GR) enzymes. SOD activity was assayed by determining its ability to inhibit the photochemical reduction of NBT as described (Beauchamp and Fridovic, 1971). G-POD activity was assessed using guaiacol as a substrate (Cakmak and Marschner, 1992). APX activity in the tomato leaves was determined as described (Nakano and Asada, 1981). CAT activity was measured according to the method as described (Cakmak and Marschner, 1992). GR activity was measured and calculated as described (Foyer and Halliwell, 1976). For the assessment of H^+^-ATPase activity, 0.3 g of fresh sample was homogenized in 50 mM Tris-HCl buffer. Then, the release of Pi was measured using an activity assay kit (Jiancheng Bio Co., Nanjing, Jiangsu, China) as described (Ahmed et al., 2013).

### Extraction and Quantification of Endogenous Melatonin by HPLC

To measure the endogenous plant melatonin concentration, a direct sampling extraction procedure was used as described (Arnao and Hernandez-Ruiz, 2009a) with modifications. Fresh leaf or root samples (0.3 g) were cut into small sections and placed into a 15 mL tube containing 6 mL of chloroform, which was shaken overnight at 4°C in the dark. The sections were extracted again using 2 mL of chloroform. The supernatant was transferred to a C^18^ solid-phase extraction (SPE) cartridge (Waters, Milford, MA, USA) for the purification of melatonin. The extract was then concentrated to dryness under nitrogen. The residue was dissolved in 2 mL of the mobile phase for HPLC analysis as described (Korkmaz et al., 2014).

### Extraction, Derivatization, and Separation of Thiol Compounds by HPLC

For thiol extraction, fresh leaf, or root samples (1 g) were homogenized with liquid nitrogen, and then 1 mL of extraction buffer containing 6.3 mM diethylene triamine pentaacetic acid (DTPA) and 0.1% (v/v) trifluoroacetic acid (TFA) was added. The supernatant was collected by centrifugation at 12,000 *g* for 10 min at 4°C. The derivatization of the thiol compounds GSH and PCs was analyzed with monobromobimane (mBBr) according to the method as described (Minocha et al., 2008). The derivative samples were filtered with 0.45 μm nylon syringe filters and stored at -20°C for up to 1 week for HPLC analyses. The thiol compounds were separated by HPLC using a fluorescence detector and a Phenomenex Synergi Hydro-RP C^18^ column (4 μm particle size, 100 mm × 4.6 mm) and a C^18^ SecurityGuard^TM^ (5 μm, 4 mm × 3 mm) cartridge guard column. The excitation and emission wavelengths were set at 380 and 470 nm, respectively, as described (Minocha et al., 2008).

### Histochemical Localization of Cd

The histochemical detection of Cd in both the leaf and root tissues was performed using a staining-based method (Seregin and Ivanov, 1997). This method involves staining with dithizone, a reagent that produces an insoluble red salt (Cd-dithizonate) in the presence of Cd. Plant samples were removed from the glutaraldehyde solution, and cross sections were taken from the root tips or the leaf veins. The sections were reacted with 2–3 drops of dithizone solution for 15 min, and then they were examined under a light microscope (BX61; Olympus Co., Tokyo, Japan).

### Separation of Tissue Fractionations

To observe the subcellular compartmentalization of Cd, the tissues were separated into the following four fractions using differential centrifugation techniques: the cell wall (CW), vacuole (V), organelle (O), and soluble (S) fractions. All of the fractions except for the V fraction were separated as previously described (Wu et al., 2005). Cell vacuoles were separated according to the method as described (Marty and Branton, 1980). In brief, fresh (5 g) root and leaf samples were homogenized in pre-chilled extraction buffer containing 50 mM Tris-HCl (pH 7.5), 250 mM sucrose, 1.0 mM DTE (C_4_H_10_O_2_S_2_), 5.0 mM ascorbic acid and 1.0% w/v Polyclar AT PVPP, pH 7.5. The homogenate was sieved through a nylon cloth (80 μM), and the residue on the nylon cloth was washed twice with the same homogenization buffer and designated as the CW fraction. This first filtrate was centrifuged at 2,000 *g* for 10 min, and the precipitate was designated as the V fraction. The resulting supernatant solution was further centrifuged at 15,000 *g* for 30 min. The resulting pellet and supernatant were referred to as the O and S fractions, respectively. All steps were performed at 4°C.

### Determination of Cd

The different separated cell fractions or 0.20 g of homogenized dry powdered sample were digested with an HClO_4_ and HNO_3_ mixture (v/v = 1:3) at 180°C. The digested colorless liquids were washed with 2 mL of diluted HNO_3_ (distilled water/concentrated HNO_3_ = 1:1) and were then washed three times with distilled water. The liquid was collected and transferred to 50 mL volumetric flasks and diluted to a constant volume. The Cd concentrations in the different fractions and the total Cd were analyzed using an atomic absorption spectrophotometer (AA-6300; Shimadzu Co., Kyoto, Japan; Wu et al., 2005).

### Extraction of Total RNA and Quantitative Real-Time PCR (qRT-PCR) Analyses

Total RNA extraction from the tomato leaf and root tissues was performed with an RNA extraction kit (Tiangen, Shanghai, China). A 1 μg aliquot of total RNA was reverse-transcribed to generate cDNA using a ReverTra Ace qPCR RT Kit (Toyobo, Japan), following the manufacturer’s instructions. qRT-PCR was performed with SYBR Green PCR Master Mix (Takara, Tokyo, Japan) using an ABI Step One Plus real-time machine (Eppendorf, USA) under the default thermal cycling conditions with an added melting curve. The gene-specific primers used for qRT-PCR were designed based on the mRNA or expressed sequence tag (EST) sequences of the corresponding genes (Supplementary Table S1). The PCR conditions consisted of denaturation at 95°C for 3 min, followed by 40 cycles of denaturation at 95°C for 30 s, annealing at 58°C for 30 s, and extension at 72°C for 30 s. Relative transcript levels were calculated as described by Livak and Schmittgen (2001).

### Statistical Analysis

Data were subjected to ANOVA and presented as the mean value for each treatment. Statistical analyses were performed using Data Processing System (DPS) statistical software package. A Tukey’s test (*P* < 0.05) was performed to evaluate the treatment effects. Each treatment value is the average of four to six replicates.

## Results

### Treatment with Cd Induces Melatonin Biosynthesis

**FIGURE 1:**
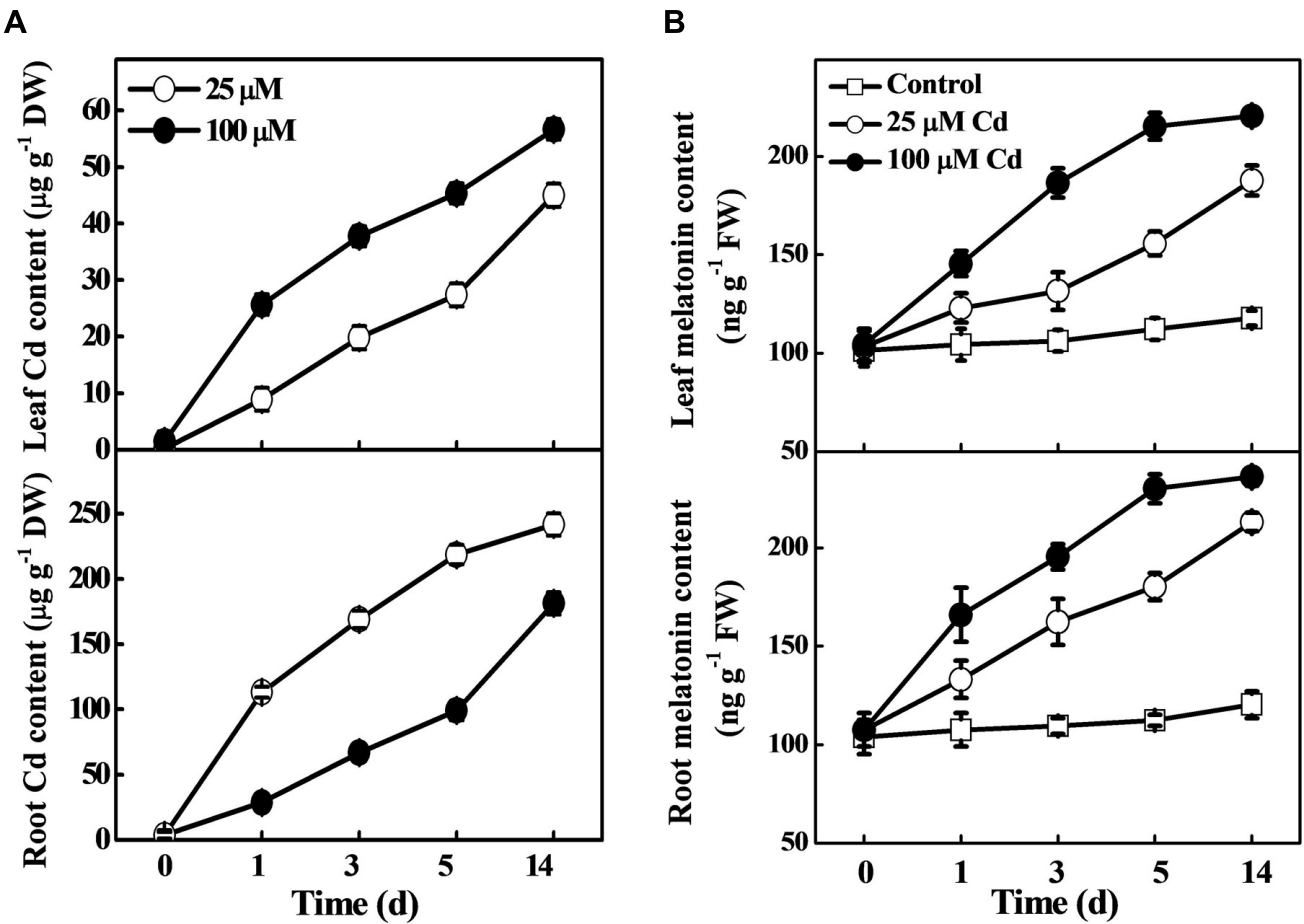
**Accumulation of cadmium (Cd) and melatonin in tomato leaves and roots as influenced by exogenous Cd treatment**. Tomato seedlings at the four-leaf stage were treated with 25 and 100 μM Cd in hydroponics. Endogenous contents of Cd **(A)** and melatonin **(B)** were determined at the indicated time points. The data are the means of four replicates, where vertical bars indicate standard errors. DW, dry weight; FW, fresh weight.

To examine the effect of ecotoxic Cd on melatonin biosynthesis, Cd uptake and translocation, we determined the concentrations of melatonin and Cd in roots and leaves of tomato plants at various time-points. As shown in **Figure 1A**, both the low (25 μM) and high (100 μM) concentration of Cd induced a substantial increase in Cd accumulation in tomato leaves and roots. In parallel with the Cd content, melatonin accumulation was gradually increased over time after Cd treatment. Notably, high Cd treatment induced high accumulation of melatonin as compared with low Cd treatment. Meanwhile, under optimum conditions, the melatonin concentration remained almost stable in both leaves and roots (**Figure 1B**). Eventually, the Cd-treated plants displayed approximately 59.6/77.2% and 87.4/96.3% increased melatonin concentration in leaves/roots than those in control under the low and high Cd treatments, respectively at 14 days (**Figure 1B**). These results confirmed that exogenous Cd could induce endogenous melatonin accumulation in leaves and roots of tomato plants.

### Effects of Exogenous Melatonin on Plant Growth, Photosynthesis, and Biomass Accumulation under Cd Stress

**FIGURE 2:**
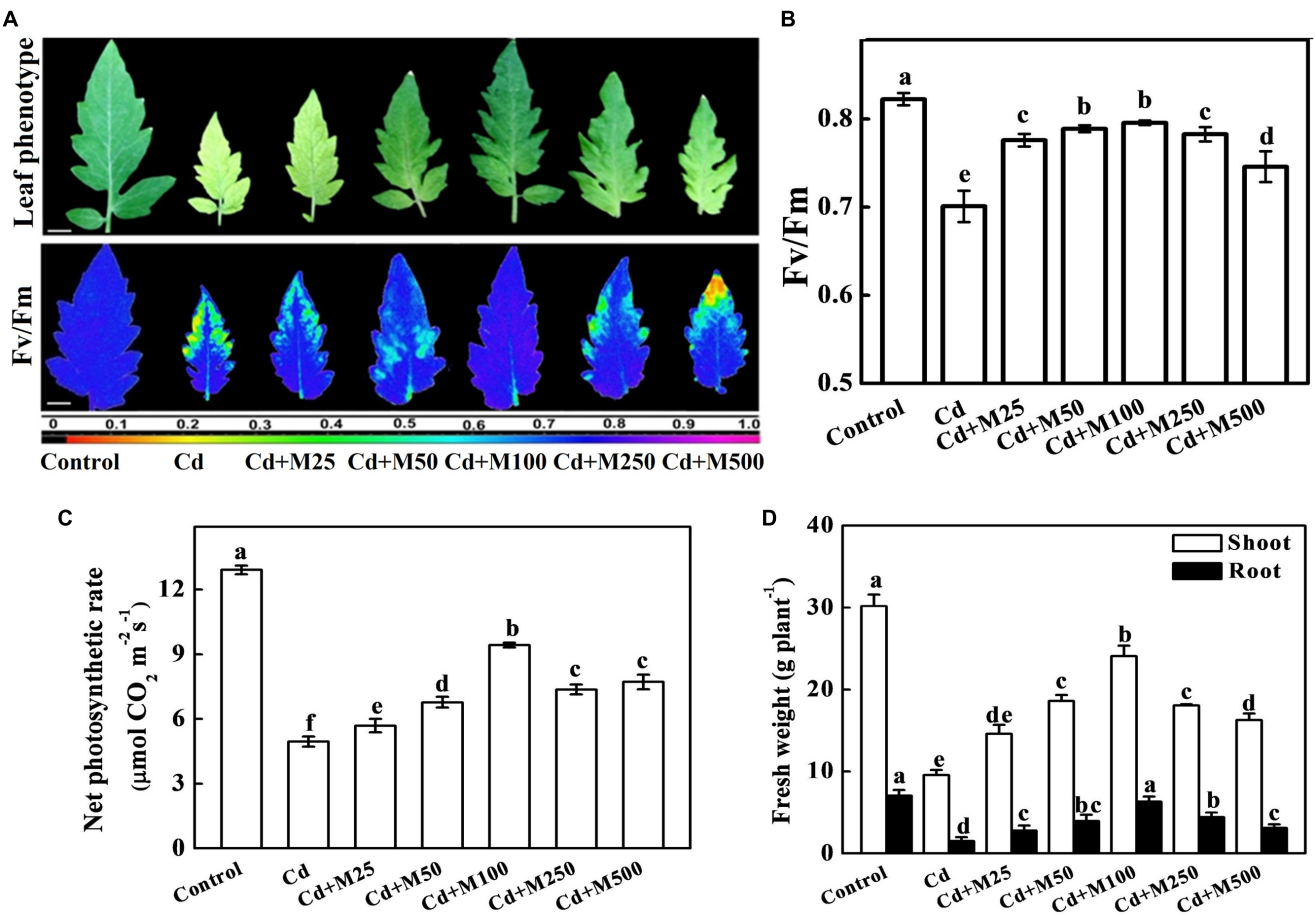
**Effects of melatonin on photochemical activity, photosynthetic capacity and biomass accumulation in tomato under Cd stress. (A)** Images of leaf phenotype(upper panel) and the maximal photochemical efficiency of PSII (Fv/Fm, lower panel), **(B)** Fv/Fm values, **(C)** net photosynthetic rate, and **(D)** Shoot and root fresh weights in tomato plants after 14 days long Cd (100 μM) stress. The data shown here are the averages of four replicates, with the standard errors indicated by the vertical bars. Means denoted by the same letters did not significantly differ at a *P* < 0.05, according to Tukey’s test. M25, 25 μM melatonin; M50, 50 μM melatonin; M100, 100 μM melatonin; M250, 250 μM melatonin; M500, 500 μM melatonin. False color code depicts at the bottom of **(A)** ranges vertically from 0.0 (black) to 1.0 (purple). Horizontal bars = 1.0 cm.

To investigate the role of exogenous melatonin in Cd tolerance, we first focused on the phenotypic changes that occurred in the tomato plants. As shown in **Figure 2**, plants treated with only Cd displayed severe leaf chlorosis, and decreased *Fv/Fm* (18.6%), *Pn* (62.1%), and fresh biomass (67.2%) when compared with that in control. In contrast, almost all doses of melatonin significantly reversed deleterious effects of Cd. Among the tested concentrations of melatonin, moderate concentration particularly 100 μM melatonin showed profound ameliorative effect as evident by 13.5 and 90.4% increased *Fv/Fm* and *Pn*, respectively compared with Cd alone treatment (**Figures 2B,C**). Similarly, Cd-induced reductions in the fresh weights of both shoot and root were significantly alleviated by the treatments with moderate concentrations of exogenous melatonin (**Figure 2D**). Considering the studied phenotypes, the order of efficiency for the melatonin concentrations in achieving positive effects against Cd was as follows: 100 > 50 > 250 > 500 > 25 μM.

### Melatonin Protects Plants from Cd-Induced Oxidative Damage

**FIGURE 3:**
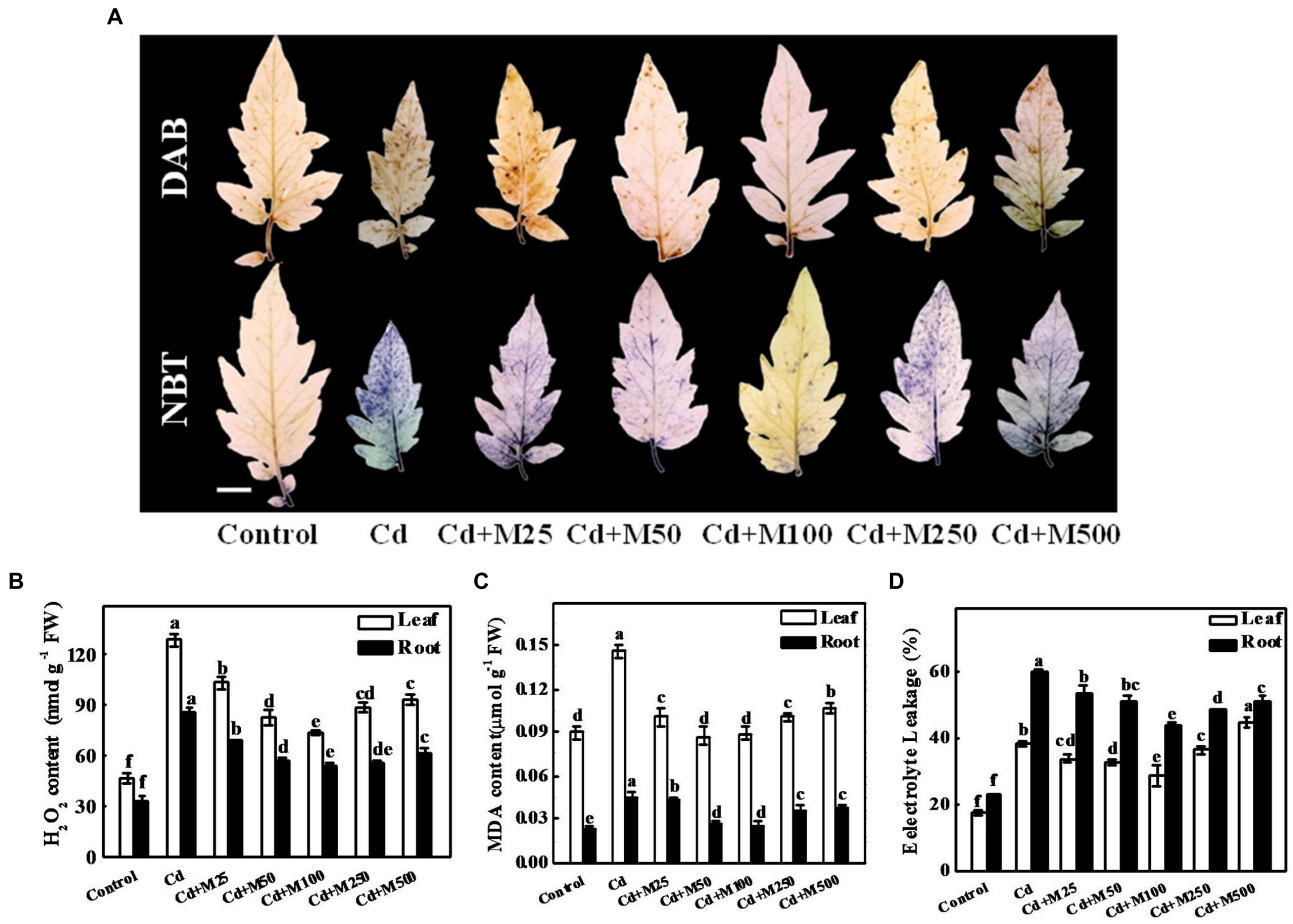
**Effects of melatonin on ROS accumulation, lipid peroxidation, and membrane integrity after 14 days long Cd stress. (A)** The *in situ* detection of H_2_O_2_ (upper panel) and $O_{2}^{•-}$ (lower panel) in tomato leaves. Bar = 1.0 cm, **(B)** H_2_O_2_ concentrations in tomato leaves and roots, **(C)** MDA concentrations in tomato leaves and roots, and **(D)** Electrolyte leakage from tomato leaves and roots. Accumulation of H_2_O_2_ and $O_{2}^{•-}$ in leaves was visually detected by staining with 3, 3-diaminobenzidine (DAB) and nitroblue tetrazolium (NBT), respectively. The data shown are the averages of four replicates, with the standard errors indicated by the vertical bars. The means denoted by the same letter within the same color histograms did not significantly differ at a *P* < 0.05, according to Tukey’s test. Cd, 100 μM cadmium; M25, 25 μM melatonin; M50, 50 μM melatonin; M100, 100 μM melatonin; M250, 250 μM melatonin; M500, 500 μM melatonin; FW, fresh weight.

Results from histochemical study showed that the ROS (H_2_O_2_ and $O_{2}^{•-}$) concentrations remained almost constant in leaves under control conditions; however, Cd treatment caused substantial increases in ROS accumulation (**Figures 3A,B**). Strikingly, melatonin application decreased the excess ROS accumulation under Cd stress (**Figures 3A,B**). The 100 μM melatonin treatment caused the greatest reduction in ROS accumulation resulting in 43.1/35.9% decreased H_2_O_2_ in the tomato leaves/roots under Cd stress (**Figure 3B**). The MDA concentrations and EL in leaves/roots were increased by approximately 112/139.2% and 118.5/159.3% in the leaf/root of Cd-treated plants compared with the control plants (**Figures 3C,D**). In line with the effect of melatonin on ROS accumulation, melatonin treatments prevented the Cd-induced increases in EL and MDA to a varying degree (**Figures 3C,D**). We also quantified the endogenous content of melatonin under different treatments (Supplementary Figure S1). It appears that exogenous melatonin has strong effect on endogenous melatonin accumulation under Cd stress, while greatest effect accounts for 100 μM melatonin. These results suggest that the application of melatonin induces endogenous melatonin content to mitigate Cd phytotoxicity through minimizing Cd-induced oxidative stress in the tomato plants.

### Melatonin Stimulates Activities of Antioxidant Enzyme and H^+^-ATPase to Confer Cd Tolerance

**FIGURE 4:**
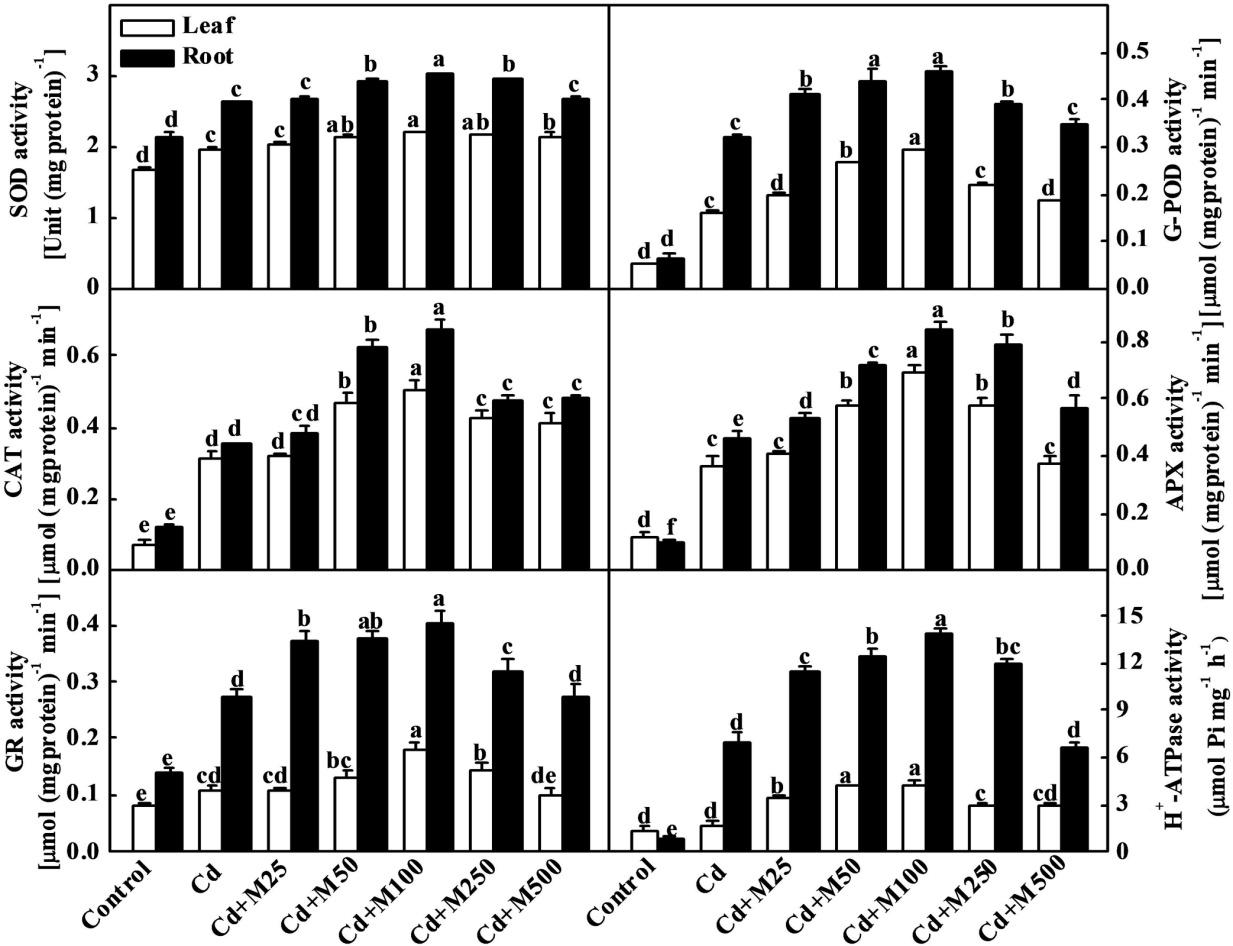
**Activities of antioxidant enzymes and H^+^-ATPase in tomato leaves and roots as influenced by melatonin and Cd treatments.** Tomato seedlings at the four-leaf stage were subjected to 100 μM Cd in hydroponics for 14 days. Meanwhile, foliar portion was sprayed with various concentrations of melatonin at every 5 days. The data shown here are the averages of four replicates, with the standard errors indicated by the vertical bars. The means denoted by the same letter did not significantly differ at a *P* < 0.05, according to Tukey’s test. Cd, 100 μM cadmium; M25, 25 μM melatonin; M50, 50 μM melatonin; M100, 100 μM melatonin; M250, 250 μM melatonin; M500, 500 μM melatonin.

To investigate the role of melatonin in the regulation of the plant antioxidative system under Cd stress, we assessed the activities of SOD, G-POD, CAT, and APX. Cd treatment induced the activities of antioxidant enzymes both in leaves and roots (**Figure 4**). Interestingly, the exogenous application of different concentrations of melatonin induced further increases in the activities of these enzymes at various degrees. Melatonin treatment at 100 μM concentration showed the best efficiency in this regard. The activities of SOD, G-POD, CAT, APX, and GR were increased by approximately 19.5/15.5%, 84.5/42.8%, 66.6/91.4%, 86.5/82.6%, and 80.2/52.3%, respectively, in leaves/roots under Cd+M100 treatment compared with Cd alone (**Figure 4**). Likewise, melatonin treatment induced the activity of plasma membrane H^+^-ATPase which acts as a primary transporter for pumping protons out of the cell (Morsomme and Boutry, 2000). Although Cd induced a 7.1-fold increase in H^+^-ATPase activity in roots, there was no significant difference in H^+^-ATPase activity in leaves between the Cd-treated and control plants (**Figure 4**). Meanwhile, compared with Cd alone treatment, H^+^-ATPase activity was further induced by combined treatment of melatonin and Cd (**Figure 4**). These results provide evidence that melatonin-induced alleviation of oxidative stress is associated with the up-regulation of the activities of antioxidant enzymes and H^+^-ATPase in the leaves and roots of tomato plants.

### Melatonin Promotes Cd Sequestration in Tomato Plants

**FIGURE 5:**
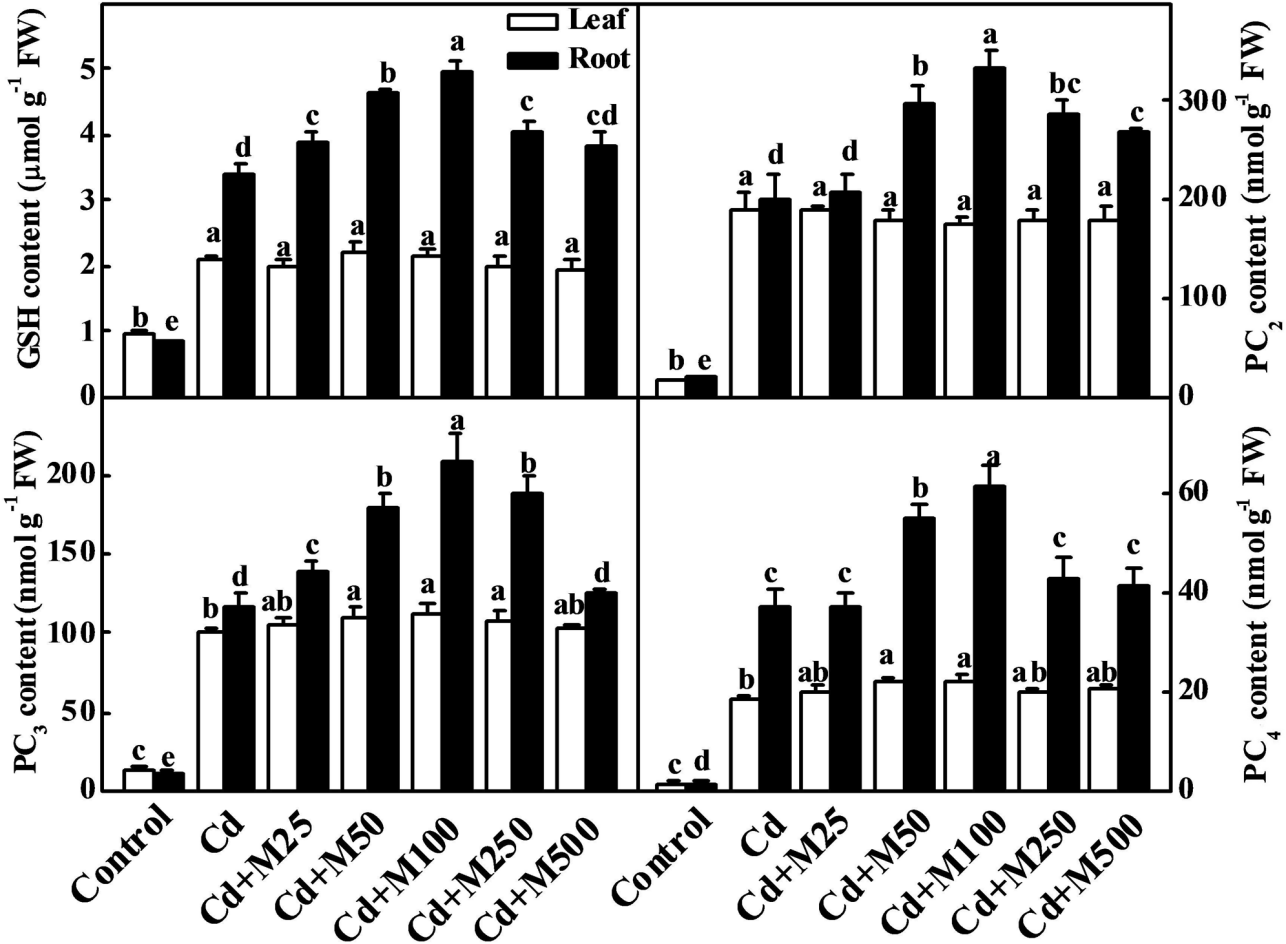
**Effects of melatonin on GSH and PCs concentrations in tomato leaves and roots after 14 days long cadmium stress.** The data shown here are the averages of four replicates, with the standard errors indicated by the vertical bars. The means denoted by the same letter within the same color histograms did not significantly differ at a *P* < 0.05, according to Tukey’s test. Cd, 100 μM cadmium; M25, 25 μM melatonin; M50, 50 μM melatonin; M100, 100 μM melatonin; M250, 250 μM melatonin; M500, 500 μM melatonin; FW, fresh weight.

To determine the role of melatonin in the biosynthesis of intracellular chelating compounds, we examined the concentrations of various PCs (PC_2_, PC_3_, and PC_4_) and GSH (precursor of PCs synthesis) under Cd stress (**Figure 5**). Cd treatment significantly increased the endogenous concentrations of PC_2_, PC_3_, PC_4_, and GSH by 9.4/8.9-fold, 7.9/10.0-fold, 14.8/28.7-fold, and 1.2/3.0-fold in leaves/roots, respectively (**Figure 5**). Interestingly, the treatments with the different concentrations of exogenous melatonin further increased the levels of these thiol compounds in roots under Cd stress (**Figure 5**). The levels of GSH, PC_2_, PC_3_, and PC_4_ were increased by 46.0, 67.8, 80.7, and 66.5%, preferentially in roots following the 100 μM melatonin treatment under Cd stress (**Figure 5**).

**FIGURE 6:**
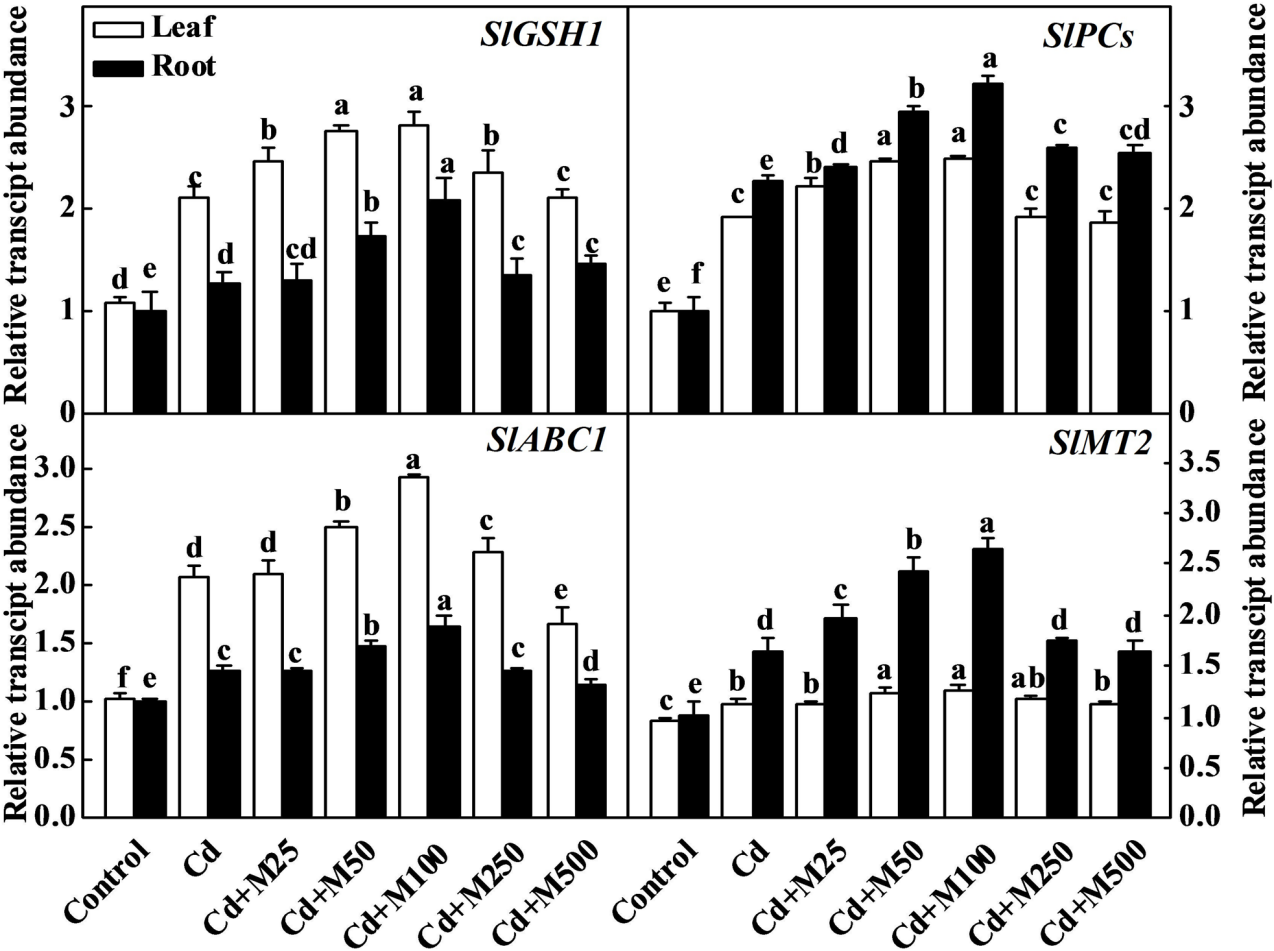
**Expression genes related to thiol compound biosynthesis in tomato leaves and roots following 14 days cadmium stress as influenced by melatonin treatments**. The data shown here are the averages of four replicates, with the standard errors indicated by the vertical bars. The means denoted by the same letter within the same color histograms did not significantly differ at a *P* < 0.05, according to Tukey’s test. Cd, 100 μM cadmium; M25, 25 μM melatonin; M50, 50 μM melatonin; M100, 100 μM melatonin; M250, 250 μM melatonin; M500, 500 μM melatonin; FW, fresh weight.

To further understand the molecular mechanism of melatonin-induced Cd detoxification, we analyzed the expression of the *SlGSH1, SlPCs, SlMT2*, and *SlABC1* genes, which are responsible for the synthesis of GSH, PCs, and metallothionein (Whitelaw et al., 1997; Wang et al., 2010; Ahammed et al., 2013; Patade et al., 2013), and ABC transporter that transports Cd-PC complex to vacuole (Hall, 2002; Park et al., 2012). The transcript levels of *SlGSH1, SlPCS, SlMT2*, and *SlABC1* were all induced by Cd stress (**Figure 6**). Importantly, exogenous melatonin further increased the transcript levels of those genes, and the 100 μM melatonin treatment was the most effective in this regard. The expression levels of *SlGSH1, SlPCS, SlMT2*, and *SlABC1* were increased by approximately 1.4/1.7-fold, 1.3/1.5-fold, 1.2/1.7-fold, and 1.5/1.4-fold in the leaves/roots of the plants treated with 100 μM melatonin compared with those in the leaves/roots of the non-melatonin-treated plants under Cd stress (**Figure 6**). These observations suggest that melatonin might be involved in the biosynthesis of metal chelating compounds and transport of Cd during Cd stress.

### Role of Melatonin in the Uptake, Subcellular Distribution, and Localization of Cd in Plant Cells

**FIGURE 7:**
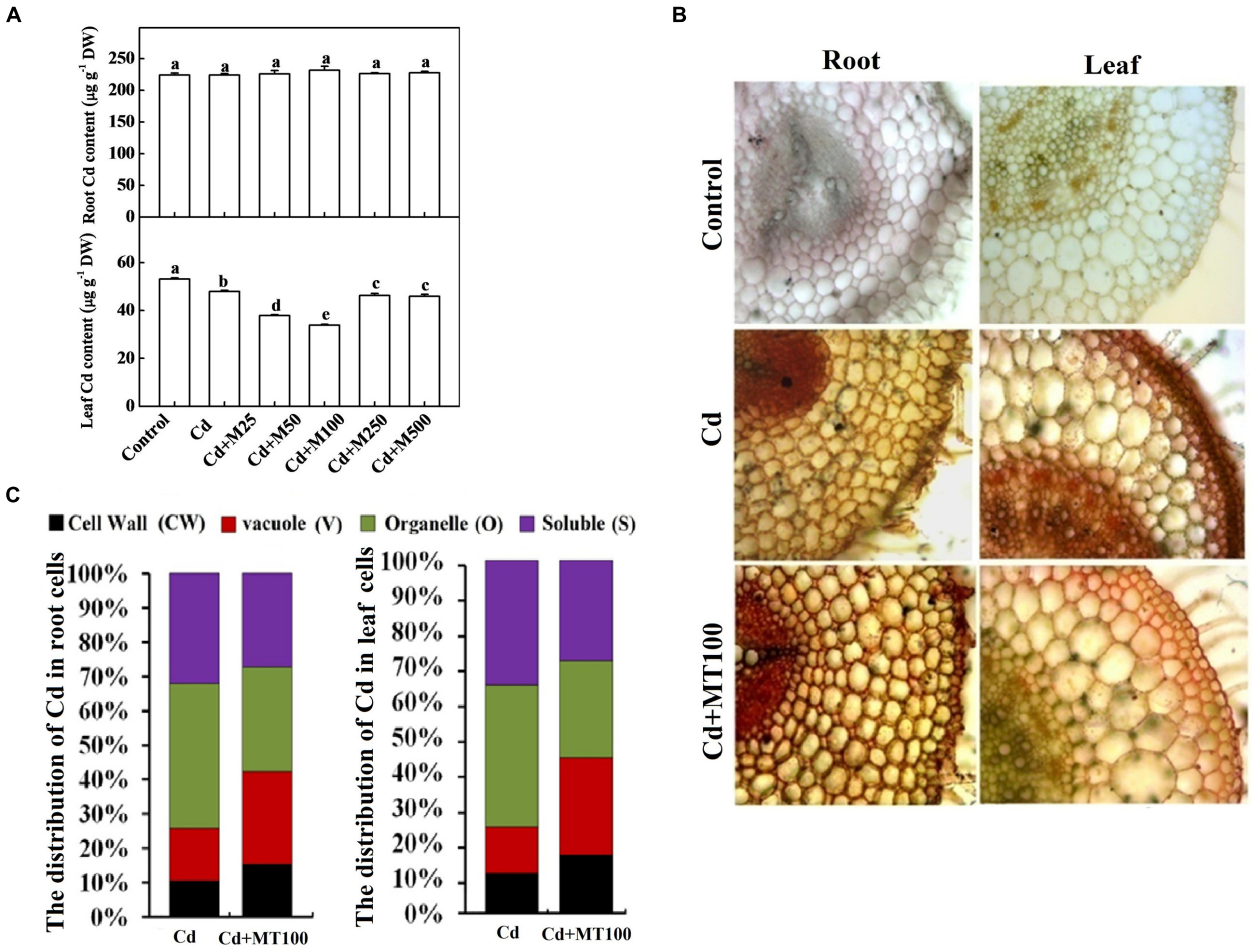
**The accumulation of cadmium in tomato plants and its subcellular distribution following 14 days long Cd stress as influenced by melatonin treatments. (A)** Cd concentrations in tomato roots and leaves. **(B)** The *in situ* detection of Cd in tomato roots and leaves using a dithizone staining-based histochemical method. **(C)** The distribution of Cd in different subcellular compartments. The data shown in **(A)**, are the averages of four replicates, with the standard errors indicated by the vertical bars. The means denoted by the same letter did not significantly differ at a *P* < 0.05, according to Tukey’s test. Cd, 100 μM cadmium; M25, 25 μM melatonin; M50, 50 μM melatonin; M100, 100 μM melatonin; M250, 250 μM melatonin; M500, 500 μM melatonin; DW, dry weight.

To understand the effects of melatonin on Cd uptake and its cellular distribution in tomato cells, we assessed the total Cd content, subcellular distribution and localization in leaf and root tissues. The Cd concentrations were approximately 224.19 and 54.16 μg (g DW)^-1^ in roots and leaves, respectively, after 14-days long Cd treatment (**Figure 7A**). Interestingly, Cd accumulation was not altered in roots but was significantly reduced in leaves with the melatonin treatments (**Figure 7A**). Treatment with 100 μM melatonin resulted in the greatest reduction in the leaf Cd concentration which was about 39.15% below the Cd alone treatment (**Figure 7A**). We also visualized the localization of Cd in root and leaf tissues using a dithizone staining-based histochemical method. As shown in **Figure 7B**, compared with leaf tissue, intensive Cd accumulation was observed in root steles regardless of the melatonin treatment. Similarly, the less intensive brown color deposits in leaf tissues indicated lower Cd content in leaf as compared with root. In line with quantitative data, visualization of Cd in Cd + 100 μM melatonin treatment showed less Cd accumulation in leaf tissues when compared with Cd alone treatment (**Figure 7B**).

To examine the subcellular distribution of Cd as influenced by exogenous melatonin treatments, we quantified the Cd content in various cellular compartments of leaf and root tissues. We found that under Cd alone treatment, the Cd levels in the cell wall (CW), vacuole (V), organelle (O), and soluble (S) fractions were approximately 10.6/11.3%, 15.3/13.1%, 42.1/40.2%, 32.0/35.4% of total Cd content in the root/leaf cells, respectively (**Figure 7C**). Importantly, treatment with 100 μM melatonin altered the subcellular distributions of Cd in both root and leaf cells. The Cd levels in the CW and V fractions, where Cd is preferentially sequestered to minimize cellular toxicity, were increased by 25.9/24.3% and 42.1/44.1% in the root/leaf cells, respectively after melatonin treatment (**Figure 7C**). Meanwhile, the Cd levels in the O and S fractions, where Cd is mostly free and highly toxic, were decreased by 74.1/75.6% and 57.8/55.9% in the root/leaf cells, respectively after melatonin treatment (**Figure 7C**). These findings indicate a critical role of melatonin in promoting Cd immobilization for minimizing cellular toxicity.

## Discussion

In the present work, we elucidated some unique functions of melatonin in response to Cd stress in tomato, an important horticultural crop and model plant system as well. We noticed that endogenous melatonin could be induced by both low and high concentrations of Cd (**Figure 1**). Notably, exogenous application of melatonin significantly improved plant tolerance to Cd by enhancing the antioxidant capacity, PCs biosynthesis, and compartmentalization of Cd in cell walls and vacuoles (**Figures 2–7**). Besides, melatonin restricted Cd transport from root to shoot in order to protect photosynthetic apparatus from Cd-induced damages. We propose that, in addition to direct function of melatonin as antioxidant and metal chelator, melatonin-induced biosynthesis of phytochelatins and subsequent compartmentalization of Cd in cell wall and vacuole might play a critical role to confer Cd tolerance in tomato.

Melatonin is a major animal hormone involved in regulating seasonal reproductive function and modulating circadian rhythms. In recent years, a growing body of evidence has suggested that it plays a significant role in plant responses to environmental stimuli, including biotic and abiotic stresses (Park et al., 2013; Zhang et al., 2013; Bajwa et al., 2014; Lee et al., 2014). Oxidative stress resulted from various forms of environmental extremes has been reported to affect the endogenous level of melatonin in a range of plant species (Arnao and Hernandez-Ruiz, 2009b). Additionally, exogenous application of melatonin was found effective to protect plant cell from oxidative damage (Zhang et al., 2015). In the present study, exposure of tomato seedlings to low and high concentrations of Cd induced substantial increases in endogenous melatonin concentrations gradually over time. However, such increase in endogenous melatonin was unable to ameliorate oxidative stress possibly due to insufficient ROS scavenging by melatonin in parallel with Cd-induced ROS generation. Nevertheless, induction of endogenous melatonin by Cd may indicate an adaptive response in plants as melatonin is well-recognized as a ubiquitous signal molecule (Arnao and Hernández-Ruiz, 2014; Byeon et al., 2015). In contrast, exogenous application of melatonin efficiently ameliorate Cd-induced oxidative stress as characterized by low ROS accumulation, reduced MDA content and decreased EL percentage. We also noticed that exogenous melatonin greatly induced endogenous melatonin content in a concentration-dependent manner. The highest level of melatonin was recorded in 100 μM melatonin + Cd treatment followed by a decreasing trend with the increase in exogenous melatonin concentration (Supplementary Figure S1). This observation confirmed that exogenous melatonin could stimulate endogenous melatonin level in a dose dependent manner to confer Cd tolerance. It is worth mentioning that low and high concentration of melatonin showed significantly different roles in regulating plant growth and development even in the same species, which support our current observation (Zhang et al., 2015). By virtue, melatonin can cross cell membrane easily and enter subcellular compartments. Given that melatonin is a ubiquitous antioxidant and thus it might be plausible that exogenous melatonin functions as an antioxidant at the initial stage, which is strictly supply dependent (Poeggeler et al., 1996; Tan et al., 2000). As the frequency of melatonin application was sparse in our current study, exogenous melatonin might not suffice proper neutralization of ROS generated from continuous Cd exposure, indicating some other mechanisms involved in melatonin-mediated Cd stress tolerance. It is quite possible that exogenous melatonin may trigger endogenous melatonin biosynthesis which then serves as antioxidant and also regulates other defense pathways for environmental adaptation.

Recent studies have revealed that pre-sowing seed treatment with melatonin protects red cabbage seedlings against toxic Cu^2+^ (Posmyk et al., 2008), and the supplementation with exogenous melatonin alleviates Cd-induced stress in algae (Tal et al., 2011). In cucumber seedlings, melatonin treatment decreased chlorophyll degradation and enhanced the photosynthetic rate and antioxidant potential, and thereby ameliorating the deleterious effects of water stress (Zhang et al., 2013). Consistent with these findings, we also found that melatonin increased the chlorophylls contents (data not shown), photosynthetic capacity and biomass accumulation in both non-stressed and Cd-stressed plants. (**Figure 2**; Supplementary Figure S2). It is speculated that melatonin increases photosynthetic efficiency through an unusual biostimulatory pathway by improving the efficiency of photosystem II under both dark and light conditions in plants (Wang et al., 2013). Serving as a signal, melatonin can activate the antioxidant system to promote the scavenging of stress-induced ROS (Tal et al., 2011; Park et al., 2013). Accordingly, melatonin activated SOD, G-POD, APX, CAT, and GR in the present study, which might partially contribute to ROS scavenging under Cd stress (**Figure 4**). Taken together, it is quite likely that melatonin acts as a signal molecule to trigger defense systems, such as the antioxidant system, resulting in the alleviation of oxidative stress.

Phytochelatins are synthesized from GSH through an enzymatic pathway, and they chelate metal ions with the -SH groups of cysteine residues (Hall, 2002). The current study showed that thiol compounds, including GSH and PCs, accumulated under Cd stress in tomato leaves and roots, were further induced in roots by exogenous melatonin treatment (**Figure 5**). Melatonin also increased GR activity required to maintain the GSH/GSSG (oxidized GSH) ratio in tomato plants under Cd stress (**Figure 4**). Recently, Wang et al. (2012) have found that melatonin increases the GSH concentration to delay the senescence of apple leaves by stimulating the activity of γ-glutamylcysteine. The expression levels of the *SlGSH1* and *SlPCS* genes, which are responsible for the biosyntheses of GSH and PCs in tomato plants, respectively, were increased by Cd. Exogenous melatonin treatment further upregulated expression of *SlGSH1* and *SlPCS* (**Figure 6**). Although expression of those genes and GR activity were increased in the melatonin-treated leaves under Cd stress (**Figure 6**), the concentration of GSH in leaves remained unaltered (**Figure 5**). Ben Ammar et al. (2008) reported that a high level of PC synthesis in roots was coupled to a decrease in the GSH concentration in leaves which might partially support our current observation. It seems that post-transcriptional events might be involved in maintaining GSH and PCs concentration in tomato leaves.

In plant cells, the toxicity of free metal ions can be decreased by their reactions with metal ligands, including proteins, polysaccharides, and organic acids (Hall, 2002). Nevertheless, the fraction of free metal ions that are not chelated can be transferred to metabolic organelles, such as chloroplasts, mitochondria, and nuclei. These free metal ions cause malfunctions of cellular organelles and cell damage (Zeng et al., 2011). Several studies have revealed that decreased translocation of Cd from roots to shoots is an effective barrier which promotes Cd sequestration in the cortex and endodermis of roots (Akhter et al., 2012, 2014). In our study, although melatonin did not affect the Cd content in root, it (50 and 100 μM) significantly reduced the leaf Cd content, suggesting that melatonin might induce barrier for preventing translocation of Cd from root to shoot (**Figures 7A,B**). We also noticed that exogenous melatonin treatment greatly improved immobilization of Cd in the cell wall and vacuoles that substantially minimized Cd toxicity (**Figure 7C**). Cd immobilization may be correlated with the melatonin-induced biosynthesis of thiol compounds (**Figure 5**; Ben Ammar et al., 2008). Given that melatonin restricted Cd translocation from root to shoot, it might facilitate healthy cellular atmosphere for normal functioning in leaves. As leaves accumulated less Cd following melatonin treatment, demand for GSH and PCs in leaves was comparatively low for metal detoxification, while roots appeared as major site for Cd detoxification staying in direct contact. It seemed logical that the higher thiol-peptide levels in roots of melatonin-treated plants might act as a sink for thiol-reactive metal and thus these plants could sequester higher levels of Cd. All of these results indicate that exogenous melatonin increases the proportion of immobilized Cd through cell wall binding and vacuole sequestration, thereby increasing the detoxification of this metal (Zeng et al., 2011).

Cadmium competes for the same transport systems with mineral nutrients, and it alters plasma membrane stability and the ionic balance (Llamas et al., 2000). Plasma membrane H^+^-ATPase transports ions and organic compounds across the plasma membrane (Morsomme and Boutry, 2000). Melatonin can be transformed into 5-methoxytryptamine and thus stimulating H^+^-ATPase activity in plants (Hardeland et al., 1995). Nevertheless, melatonin also protects the plasma membrane lipid environment by preventing Cd-induced ROS generation (Tan et al., 2012) and maintaining increased levels of antioxidants (Zhang et al., 2013). In line with previous reports, the current study showed that melatonin induced H^+^-ATPase activity under Cd stress (**Figure 4**), which might have significant role in maintaining plasma membrane stability and EL to confer Cd tolerance (**Figure 3D**).

Survival of plants under polluted environments largely depends on plants’ ability to sequester and/or detoxify toxic pollutants such as Cd. This study showed that both exogenous melatonin and Cd stress could induce endogenous melatonin accumulation. However, exogenous melatonin had complex and influential effects on plant growth, ROS scavenging, antioxidant potential, thiol compound biosynthesis and Cd immobilization in cell walls and vacuoles under Cd stress. Even though, exogenous melatonin had no effect on root Cd content, it significantly decreased leaf Cd content, indicating its potential role in regulating Cd translocation. Therefore, these findings can be implicated for developing new strategies to produce safe food in an eco-friendly manner particularly in marginal areas where Cd contamination is a limiting factor for crop production. Further studies are needed to provide genetic evidence in support of the involvement of melatonin in Cd detoxification and to elucidate the subsequent signaling cascades in plants.

## Conflict of Interest Statement

The authors declare that the research was conducted in the absence of any commercial or financial relationships that could be construed as a potential conflict of interest.
