# Corrigendum: Melatonin mitigates cadmium phytotoxicity through modulation of phytochelatins biosynthesis, vacuolar sequestration, and antioxidant potential in *Solanum lycopersicum* L

**DOI:** 10.3389/fpls.2024.1431121

**Published:** 2024-05-21

**Authors:** Md. Kamrul Hasan, Golam Jalal Ahammed, Lingling Yin, Kai Shi, Xiaojian Xia, Yanhong Zhou, Jingquan Yu, Jie Zhou

**Affiliations:** ^1^ Department of Horticulture, Zhejiang University, Hangzhou, China; ^2^ Zhejiang Provincial Key Laboratory of Horticultural Plant Integrative Biology, Hangzhou, China; ^3^ Key Laboratory of Horticultural Plants Growth, Development and Quality Improvement, Agricultural Ministry of China, Hangzhou, China

**Keywords:** cadmium, food safety, melatonin, phytochelatins, tomato, vacuolar sequestration

In the published article, there were a couple of errors in **Figure 3A** and **Figure 7A** as published. In **Figure 3A**, the representative images of NBT-stained leaflets (2nd row) after Cd (panel 2), Cd+M250 (panel 6) and Cd+M500 (panel 7) treatments were overwritten by redundant images, respectively. In **Figure 7A**, ‘Control’ was written in the X-axis legend mistakenly. The corrected **Figure 3** and **Figure 7** and their captions appear below.

**Figure 3:**
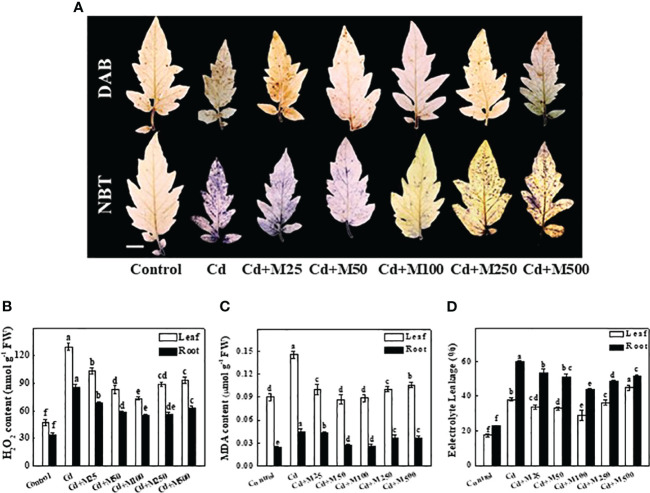
Effects of melatonin on ROS accumulation, lipid peroxidation, and membrane integrity after 14 days long Cd stress. **(A)** The *in situ* detection of H_2_O_2_ (upper panel) and O_2_^·−^ (lower panel) in tomato leaves. Bar = 1.0 cm, **(B)** H_2_O_2_ concentrations in tomato leaves and roots, **(C)** MDA concentrations in tomato leaves and roots, and **(D)** Electrolyte leakage from tomato leaves and roots. Accumulation of H_2_O_2_ and O_2_^·−^ in leaves was visually detected by staining with 3, 3-diaminobenzidine (DAB) and nitroblue tetrazolium (NBT), respectively. The data shown are the averages of four replicates, with the standard errors indicated by the vertical bars. The means denoted by the same letter within the same color histograms did not significantly differ at a *P* < 0.05, according to Tukey’s test. Cd, 100 μM cadmium; M25, 25 µM melatonin; M50, 50 µM melatonin; M100, 100 µM melatonin; M250, 250 µM melatonin; M500, 500 µM melatonin; FW, fresh weight.

**Figure 7:**
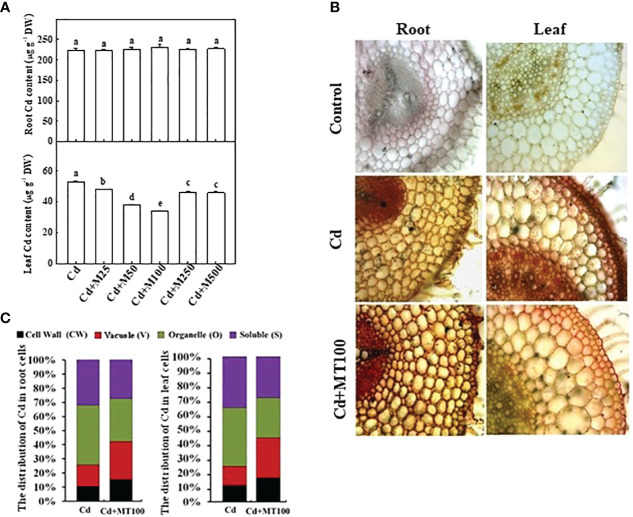
The accumulation of cadmium in tomato plants and its subcellular distribution following 14 days long Cd stress as influenced by melatonin treatments. **(A)** Cd concentrations in tomato roots and leaves. **(B)** The *in situ* detection of Cd in tomato roots and leaves using a dithizone staining-based histochemical method **(C)** The distribution of Cd in different subcellular compartments. The data shown in **(A)**, are the averages of four replicates, with the standard errors indicated by the vertical bars. The means denoted by the same letter did not significantly differ at a *P* < 0.05, according to Tukey’s test. Cd, 100 μM cadmium; M25, 25 µM melatonin; M50, 50 µM melatonin; M100, 100 µM melatonin; M250, 250 µM melatonin; M500, 500 µM melatonin; DW, dry weight.

The authors apologize for these errors and state that these do not change the scientific conclusions of the article in any way. The original article has been updated.

