# Supplementary material for: Melatonin mitigates cadmium phytotoxicity through modulation of phytochelatins biosynthesis, vacuolar sequestration, and antioxidant potential in Solanum lycopersicum L
Source: Front Plant Sci. 2015 Aug 11;6:601. doi: 10.3389/fpls.2015.00601 (PMC4531246; doi:10.3389/fpls.2015.00601)
Supplement: Supplementary file 1 [file Data_Sheet_1.DOCX]

**Supplementary Figure 1**

**Supplementary Figure 1.** Effects of melatonin application on plant endogenous melatonin content under Cd stress. Tomato seedlings at the four-leaf stage were sprayed with 0, 25, 50, 100, 250 and 500 μM melatonin (every five days) and samples were harvested after two weeks of initial melatonin treatment. The data shown are the averages of four replicates, with the standard errors indicated by the vertical bars. The means denoted by the same letter within the same color histograms did not significantly differ at a *P* < 0.05, according to Tukey’s test. Control, water; M25, 25 µM melatonin; M50, 50 µM melatonin; M100, 100 µM melatonin; M250, 250 µM melatonin; M500, 500 µM melatonin.

**Supplementary Figure 2**

**(a)**

**(b)**

**Supplementary Figure 2.** Effects of different concentrations of melatonin on (a) net photosynthetic rate and, (b) biomass accumulation in tomato. Each value is the average of four replicates, with the standard errors indicated by the vertical bars. The means denoted by the same letter within the same color histograms did not significantly differ at a *P* < 0.05, according to Tukey’s test. Control, water; M25, 25 µM melatonin; M50, 50 µM melatonin; M100, 100 µM melatonin; M250, 250 µM melatonin; M500, 500 µM melatonin.

**Supplementary Table. S1** Primers used for real time qRT-PCR assays

| **Genes** | **Primer sequences (5′-3′)** | **Accession numbers** |
| --- | --- | --- |
| *SlGSH1* (*GAMMA-GLUTAMYLCYSTEINE SYNTHETASE 1 )* | F: TTGCTTATGCATGTTGCTCA;  R: ACAACCTCGGCTACTTCGTT | Solyc08g081010.2.1 |
| *SlPCs* (*PHYTOCHELATIN SYNTHASE)* | F: GAAGGTCTGGTTTGTCGGAT;  R: CCATTCCCATTCCCATTTAC | Solyc09g072620.2.1 |
| *SlABC1* *(ABC TRANSPORTER C FAMILY MEMBER 2)* | F: CCATGGCTAGGGCTGTTTAT;  R: GTTCTCCCTTGATGCACCTT | Solyc08g006880.2.1 |
| *SlMT2* *(TYPE 2 METALLOTHIONEIN)* | F: AGCAGCACAACCACTGAGAC;  R: GGTTGCACTTGCAGTCAGAT | Solyc09g010800.2.1 |
| *Actin* (House keeping gene-internal control) | F: TGGTCGGAATGGGACAGAAG;  R: CTCAGTCAGGAGAACAGGGT | Solyc03g078400.2.1 |
